# Decline in Lung Function From Mid-to Late-Life With Central Arterial Stiffness: The Atherosclerosis Risk in Communities Study

**DOI:** 10.1177/00033197221105747

**Published:** 2022-05-27

**Authors:** Kennedy M. Peter, James R. Pike, John S. Preisser, Anna M. Kucharska-Newton, Michelle L. Meyer, Maria C. Mirabelli, Priya Palta, Timothy Hughes, Kunihiro Matsushita, Yifei Lu, Gerardo Heiss

**Affiliations:** 1 41474 University of North Carolina at Chapel Hill, Chapel Hill, NC, USA; 2 University of Kentucky – Lexington, Lexington, KY, USA; 3 25798 Emory University, Atlanta, GA, USA; 4 21611 Columbia University, New York, NY, USA; 5 12279 Wake Forest University, Winston-Salem, NC, USA; 6 25802 Johns Hopkins University, Baltimore, MD, USA

**Keywords:** pulse wave velocity, arterial stiffness, lung function, spirometry

## Abstract

We investigated the association of lung function at mid-life, later in life, and its 20-year decline, with arterial stiffness later in life. We examined 5720 Atherosclerosis Risk in Communities Study participants who attended Visits 1 (1987-1989) and 5 (2011-2013). Lung function measures were forced expiratory volume in one second (FEV_1_) and forced vital capacity (FVC), obtained at Visits 1, 2 (1990–1992), and 5. Central artery stiffness (carotid-femoral pulse wave velocity [cfPWV]) was measured at Visit 5. We evaluated associations of lung function with later-life central artery stiffness and cfPWV >75th percentile by multivariable linear and logistic regressions. Lung function at Visit 1 (FEV_1_ β: −26, 95% Confidence Interval [CI]: −48, −5; FVC β: −14, 95% CI: −32, 5) and Visit 5 (FEV_1_ β: −22, 95% CI: −46, 2; FVC β: −18, 95% CI: −38, 2) were inversely associated with cfPWV at Visit 5, and with odds of high cfPWV in fully adjusted models. Twenty-year decline in lung function was not associated with continuous or dichotomous measures of arterial stiffness (FEV_1_ β: 11, 95% CI: −46, 68; FVC β: −4, 95% CI: −52, 43). Lung function at mid-life and late-life was inversely associated with arterial stiffness in later life.

## Introduction

About 14% of US adults have an impairment in lung function, which is associated with an increased risk of cardiovascular (CV) events and CV-related mortality.^1,2^ Lung function can be affected by smoking, air pollution, and respiratory disease, among other factors, and may be linked to CV disease indirectly through these exposures or through associated mechanisms such as chronic inflammation,^3^ comorbidity, or lung-heart hemodynamic interaction.^4-6^ Poor lung function has been related to several CV risk factors such as inflammation-sensitive plasma proteins,^3^ blood pressure,^7^ type II diabetes,^8^ BMI,^9^ and presence of carotid plaques.^9^ Additionally, rapid declines in lung function were associated with incident CV events such as heart failure, stroke, and coronary heart disease in the Atherosclerosis Risk in Communities (ARIC) Study.^10^ Central arterial stiffening, characterized by remodeling of the extracellular matrix of the arterial wall resulting in changes to the hemodynamics,^11^ is also a risk factor for CV events and damage to end organs.^12,13^ While poor lung function and arterial stiffening are both demonstrably associated with cardiovascular outcomes, limited evidence suggests a relationship between poor lung function and arterial stiffness.

Poor lung function and central arterial stiffness often co-occur, but causal and temporal relationships are equivocal. Several biological mechanisms have been proposed that may connect poor lung function and arterial stiffening, including chronic systemic inflammation,^3^ hypertension,^7,13^ and diabetes.^8,14^ Associations between lung function and arterial stiffness have been well established in chronic obstructive pulmonary disease (COPD) patients, suggesting that those with COPD have stiffer arteries than those without COPD.^15^ These studies further suggest that arterial stiffness increases with increasing severity of COPD symptoms, and therefore, with poorer lung function.^16^ However, only a few studies have investigated the relationship between lung function and arterial stiffness in populations free of lung disease. Most prior studies of lung function and arterial stiffness, as measured by pulse wave velocity (PWV), have been cross sectional, and support an inverse association between lung function and arterial stiffness. The majority of cross-sectional studies assessing expiratory function, measured by forced expiratory volume in one second (FEV_1_), found poor expiratory function to be associated with greater arterial stiffness,^17-22^ while some did not.^9,23^ Other studies have assessed the cross-sectional association of lung capacity, measured by force vital capacity (FVC), and arterial stiffness, finding mixed results: some studies found poor lung capacity to be associated with stiffer arteries,^19,21-25^ while others did not.^18^

To our knowledge, only two studies have examined the relationship between lung function and central arterial stiffness prospectively, and only one in terms of lung function changes over time. One of these studies found that poor expiratory function was associated with stiffer arteries 5 years later,^17^ and the other reported that both expiratory function and lung capacity at mid-life were better predictors of later-life arterial stiffness (20 years later) than measures of lung function later in life.^19^ Only one study reported on temporal change in lung function, finding that a greater 5-year decline in expiratory function was associated with greater arterial stiffness at the end of the 5-year period.^17^ Studying prospective associations of lung function and rate of decline in lung function with arterial stiffness is thus needed to elucidate temporal relationships between lung function and arterial stiffening.

Investigating relationships of lung function at different time points and its change over time with central arterial stiffness later in life would provide insights into later-life vascular aging. Therefore, this prospective, longitudinal cohort study investigated associations of mid-life lung function, later-life lung function, and 20-year decline in lung function, with later-life central arterial stiffness in the ARIC Study.

## Methods

### Study Design and Population

The ARIC Study is a population-based, prospective cohort study investigating the etiology of atherosclerotic disease in a middle-aged population of mainly white and Black men and women.^26^ From 1987 to 1989, 15 792 participants ages 45 to 64 years were recruited from four communities in the United States: Forsyth County, NC; Jackson, MS; suburbs of Minneapolis, MN; and Washington County, MD. The initial evaluation was followed by assessments conducted during Visit 2 (1990–1992, n = 14 348), Visit 3 (1993–1995, n = 12 887), Visit 4 (1996–1998, n = 11 656), and Visit 5 (2011–2013, n = 6538). The protocol for each visit was approved by the institutional review boards at Johns Hopkins University, Wake Forest University, University of Mississippi Medical Center, the University of Minnesota, and the University of North Carolina at Chapel Hill. Written informed consent was obtained from each participant or their legally authorized representative.

Out of all 15 792 ARIC participants at baseline (Supplementary Figure 1), we excluded participants with a self-reported race other than Black or white (n = 48) and Black participants from Washington County, MD or the suburbs of Minneapolis, MN (n = 55) due to small sample sizes. To ensure a population free of lung disease, we further excluded those with self-reported asthma or chronic lung disease at baseline (n = 1638), and participants with ICD-9 codes for COPD-related hospitalizations (n = 730). Those deceased by ARIC visit 5 (n = 4038) were excluded, resulting in 9283 participants in the analytic sample. Measurement issues or missing data occurred for the following 2360 participants among the 5710 participants in our analytic sample who attended ARIC Visit 5. Very few participants (n = 8) had incomplete measures of lung function at both Visits 1 and 2. Visit 5 missing lung function and spirometry grade “F” measures were observed among 1559 participants and 73 participants, respectively. We further excluded participants with missing central arterial stiffness measures at Visit 5 (n = 370), and those who at Visit 5 had conditions that may affect the quality of the cfPWV measure (BMI ≥40 [n = 101]; evidence of major arrhythmia [n = 106]; self-reported aortic revascularization surgery [n = 32]; MN codes 8-1-2 that influenced pulse wave velocity waveforms [n = 17]; and aortic stenosis and moderate/greater aortic regurgitation [n = 35]). Lastly, we determined 59 participants to have outlying values of lung function or central arterial stiffness. This resulted in 3360 participants who met inclusion criteria and who did not have missing or mismeasured lung function and arterial stiffness.

### Lung Function

Components of lung function at mid-life and later in life included expiratory function and lung capacity. Expiratory function, or how quickly air can be expelled, was estimated using FEV_1_ in liters (L). Lung capacity, or the total volume of air that can be inhaled (L), was assessed by forced vital capacity (FVC). A lower measurement of FEV_1_ or FVC in L indicates poorer lung function. FEV_1_ and FVC were measured at ARIC Visits 1 and 2 using the Collins Survey II water-seal spirometer from the Warren E. Collins Company (Braintree, MA). At Visit 5, the SensorMedics model 1022 dry-rolling seal volume spirometer (OMI, Houston, TX) was used. The highest measure out of three acceptable spirogram trials was selected for both FVC and FEV_1_. Extensive quality control programs were in place and all lung function measurements were interpreted at a central reading site.^27,28^ Measures of FEV_1_ below .75 L or above 5.5 L were classified as outliers and removed from the complete-case analytic sample, and converted to missing for imputed datasets. The same approach was applied to measures of FVC below 1.25 L or above 7.0 L.

### Central Arterial Stiffness

Later life central arterial stiffness was approximated by carotid-femoral pulse wave velocity (cfPWV), considered the gold standard for measuring arterial stiffness.^29^ cfPWV at Visit 5 (2011-2013) was measured using the Omron VP-1000 plus system in cm/s. Carotid-femoral distance, assessed using the Rosscraft Segmometer, was calculated by subtracting (1) the distance from suprasternal notch to the recording site of the carotid artery from (2) the distance between the carotid artery and femoral artery recording sites. This distance was divided by the transit time of the pulse waveform, resulting in the final cfPWV measure. Higher cfPWV in cm/s indicates stiffer arteries. cfPWV measurement was repeated at least once and averaged to ensure data quality. Quality control programs were in place, including an ongoing overview of a random sample of study measurements by a central site with feedback to study technicians. Repeat visits were conducted for a subset of participants at each field center approximately 4–8 weeks later (n = 79; mean age 75.7 years; 46 females). The intra-class correlation coefficient and the 95% confidence interval (95% CI) for single cfPWV measurements were .70 (95% CI: .59, .81).^30^ Measurements below 500 cm/s or above 3000 cm/s were designated as biologically improbable outliers and removed from the complete-case analytic sample and set to missing for imputed datasets. cfPWV was modeled as a continuous variable and a binary variable dichotomized at the top quartile (cfPWV 1324-2855 cm/s, hereafter referred to as “high cfPWV”), comparing participants with high cfPWV to participants with non-high cfPWV at Visit 5.

### Covariates

Covariates assessed at Visit 1 included biologic sex (male or female), self-reported race (Black or white), age (years), height (cm), weight (kg), high fasting plasma glucose (fasting plasma glucose ≥126 mg/dL, hereafter referred to as “high fasting plasma glucose”), and smoking status (current, former, never). Height, weight, smoking status, and high fasting plasma glucose were assessed again at Visit 5. Height and weight were used to calculate body mass index (BMI) as kg/m^2^. Most of the Black participants in this population were recruited from Jackson, Mississippi, making study-center highly correlated with race. Therefore, race was adapted into a categorical classification of race and field center (Minnesota/white, Maryland/white, North Carolina/white, North Carolina/Black, and Mississippi/Black). Covariates were identified for inclusion in models based on prior literature and a priori scientific knowledge.

### Statistical Analysis

We produced descriptive statistics of demographic, clinical, and lung function variables stratified by quartiles of cfPWV. Changes in expiratory volume and lung capacity (in L) for descriptive statistics were calculated by subtracting FVC and FEV_1_, respectively, at Visit 5 from the corresponding measure at Visit 1. Lung function at Visit 1, 5, and its change were adjusted for age, height, race, and sex at the corresponding time by linear regression for descriptive statistics. We examined the role of missing data and loss to follow-up in potential biases by producing descriptive statistics of baseline lung function, demographic, and clinical measures by participant status at Visit 5. Participant status at Visit 5 was defined as follows: (a) those included in the complete case analysis sample who had no missing exposures or outcomes, (b) those who attended Visit 5 but had missing or inadequately measured exposure or outcome, and (c) alive participants who did not attend Visit 5.

Decline in lung function was calculated from an unadjusted linear mixed effects model that used maximum likelihood estimation, employed an unstructured variance-covariance matrix, and incorporated a random intercept and slope. Time was defined as a continuous metric cataloging the number of years since Visit 1. Subject-specific slopes were generated and scaled such that a one-unit *increase* represented a one L *decrease* over a period of 20 years. The slopes for 20-year decrease in lung function were then used as the independent variable in subsequent models.

We assessed the shape of the unadjusted exposure-outcome relationship using Akaike and Bayesian information criterion and scatterplots, and based on this assessment, we chose a linear trend. Measures of lung function, FEV1 and FVC, were modeled separately. We assessed three different temporal associations of lung function over 20 years with later-life central arterial stiffness: a prospective association (lung function at Visit 1 with cfPWV at Visit 5), a cross-sectional association (lung function and cfPWV both measured at Visit 5), and the association between the rate of 20-year change in lung function (across Visits 1, 2, and 5) with cfPWV at Visit 5. The associations of lung function at Visit 1, Visit 5, and its 20-year decline, with cfPWV at Visit 5 were modeled using multivariable linear regressions. The associations of lung function at Visits 1 and 5, and 20-year decline in lung function, with dichotomous high cfPWV were assessed using adjusted logistic regressions. Two adjustment sets were investigated in these analyses: minimally adjusted (Model 1) and fully adjusted (Model 2). Due to variation in lung function by thoracic height, Model 1 was adjusted only for height: prospective associations and change in lung function were adjusted for height at Visit 1, while cross-sectional associations were adjusted for height at Visit 5. Model 2 examined prospective associations and 20-year decline in lung function adjusted for race-center, sex, smoking status, age, time between measurements, height, BMI, and high fasting plasma glucose, all measured at Visit 1. For Model 2, cross-sectional models were adjusted for race-center, sex, and the following variables measured at Visit 5: smoking status, age, height, BMI, and high fasting plasma glucose. Two and three-way interaction terms between race-center, smoking status, and change in age were tested to improve model fit. Interactions and quadratic terms with *P*-values ≤.1 were retained in the final models.

Multivariate imputation by chained equations (MICE)^31^ was implemented to account for missing exposures and outcome (n = 2360; Supplementary Table 1). Fifteen imputed datasets were generated, exceeding the 12 imputations recommended for a two-stage analysis.^32^ In a sensitivity analysis, exposure and outcome data were additionally imputed for all participants alive at Visit 5 who did not attend the Visit to account for differential attrition (38.4% of baseline analytic sample; n = 3563; Supplementary Table 1). As another sensitivity analysis, we assessed differences in the associations by hypertension, smoking status, and high fasting plasma glucose by testing interaction terms of these characteristics with the exposure. P-values (2-sided) ≤.05 were considered statistically significant. All analyses were completed using SAS version 9.4 (SAS Institute, Cary, North Carolina, United States).

## Results

### Participant Characteristics

At baseline, participants had a mean age of 51.4 (standard deviation [SD]: 4.9) years, consisted of 59.7% females, and were 22.3% Black (Table 1). Adjusted mean (SD) FEV_1_ at Visit 1 was 3.1 (.6) L, and adjusted mean (SD) FVC was 4.0 (.8) L. Both FEV_1_ and FVC decreased by approximately 1 L over approximately 20 years. Mean cfPWV at Visit 5 was 1161.2 (302.9) cm/s. When stratified by quartiles of cfPWV at Visit 5, participants in the highest quartile of cfPWV included more men, people of Black race, people with high fasting plasma glucose, and participants who were slightly older than the quartile corresponding to the lowest quartile of cfPWV. Lung function measures adjusted for age, sex, race, and height, at Visits 1, 5, and their change, did not differ substantially by quartiles of arterial stiffness at Visit 5 (Table 1).

**Table 1.** Demographic and Health-Related Characteristics of the Complete-Case Study Population, Stratified by Quartiles of Central Arterial Stiffness at Visit 5.

		Quartiles of cfPWV			
	All participants	Quartile 1 (500–953 cm/s)	Quartile 2 (954–1117 cm/s)	Quartile 3 (1118–1323 cm/s)	Quartile 4 (1324–2855 cm/s)
	n (%)	n (%)	n (%)	n (%)	n (%)
Total	3360	839	841	841	839
Study center					
Forsyth County, NC	649 (19.3)	176 (21.0)	169 (20.1)	159 (18.9)	145 (17.3)
Jackson, MS	709 (21.1)	140 (16.7)	142 (16.9)	172 (20.5)	255 (30.4)
Suburbs of Minneapolis, MN	1054 (31.4)	281 (33.5)	309 (35.9)	269 (32.0)	202 (24.1)
Washington County, MD	948 (28.2)	242 (28.8)	228 (27.1)	241 (28.7)	237 (28.3)
Female sex	2007 (59.7)	531 (63.3)	508 (60.4)	495 (58.9)	473 (56.4)
Black (race)	749 (22.3)	150 (17.9)	156 (18.6)	180 (21.4)	263 (31.4)
Age at Visit 1, mean (SD)	51.4 (4.9)	50.0 (4.5)	50.6 (4.4)	51.8 (4.8)	53.2 (5.1)
Height (cm) at Visit 1, mean (SD)	168.5 (9.2)	167.7 (8.9)	168.6 (9.1)	168.4 (9.2)	169.2 (9.4)
Body Mass Index at Visit 1, mean (SD)	26.5 (4.1)	26.4 (4.2)	26.4 (4.1)	26.5 (4.1)	26.6 (4.2)
High fasting plasma glucose at Visit 1	130 (3.9)	11 (1.3)	14 (1.7)	35 (4.1)	70 (8.4)
Missing	21	3	9	4	5
Cigarette smoking status at Visit 1					
Current	498 (14.9)	132 (15.8)	123 (14.6)	127 (15.1)	116 (13.8)
Former	1115 (33.2)	264 (31.5)	282 (33.6)	282 (33.6)	287 (34.2)
Never	1744 (52.0)	442 (52.7)	435 (51.8)	431 (51.3)	436 (52.0)
Missing	3	1	1	1	0
FEV_1_ at Visit 1 in L,^a^ mean (SD)	3.1 (.6)	3.1 (.6)	3.1 (.6)	3.1 (.6)	3.0 (.6)
FVC at Visit 1 in L,^a^ mean (SD)	4.0 (.8)	4.0 (.8)	4.0 (.8)	4.0 (.8)	4.0 (.8)
FEV_1_ at Visit 5 in L,^b^ mean (SD)	2.2 (.5)	2.2 (.4)	2.2 (.5)	2.2 (.5)	2.1 (.5)
FVC at Visit 5 in L,^b^ mean (SD)	3.0 (.7)	3.0 (.7)	3.0 (.7)	3.0 (.7)	3.0 (.7)
Change in FEV_1_ in L,^b^ mean (SD)	−.9 (.2)	−.9 (.1)	−.9 (.2)	−.9 (.2)	−.9 (.2)
Change in FVC in L,^b^ mean (SD)	−1.0 (.2)	−1.0 (.2)	−1.0 (.2)	−1.0 (.2)	−1.0 (.2)
cfPWV at Visit 5 in cm/s, mean (SD)	1161.2 (302.9)	825.8 (104.8)	1036.3 (47.0)	1214.4 (59.0)	1568.4 (232.3)

Abbreviations: cfPWV, carotid-femoral pulse wave velocity in cm/s; FEV_1_, forced expiratory volume in 1 second in liters; FVC, forced vital capacity in liters; SD, standard deviation; L, liters.

^a^Present and adequately measured lung function values at Visit 2 were used for participants with missing or inadequately measured lung function at visit 1, adjusted for race, sex, age, and height at Visit 1.

^b^Adjusted for race, sex, age, and height at the corresponding visit.

Investigation into bias from missing data and informative attrition yielded Supplementary Table 1. Compared to the sample with complete exposure and outcome, participants who attended ARIC Visit 5 but who had missing data (n = 2360) included more men, people of Black race, participants with high fasting plasma glucose, and smokers. There were also geographical differences by study-center between these groups. When comparing participants who met inclusion criterion and who were alive at the time of ARIC Visit 5 but did not attend the visit (n = 3563), many of the same patterns described above were present and were more pronounced (Supplementary Table 1). Due to potential for selection bias from missing data and differential attrition as suggested by these results, we have presented the results only of analyses that used MICE to account for missing data and/or informative attrition. Complete-case analyses are available in the supplement (Supplementary Tables 2 and 3).

### Associations of Lung Function and its Change with Central Arterial Stiffness

When minimally adjusted for height (Model 1), 1 L greater lung function at both baseline and ARIC Visit 5 were similarly associated with lower cfPWV: a 1 L increment of FEV_1_ measure at Visit 1 was associated with −87.7 (95% CI: −105.5, −64.3) cm/s lower cfPWV at Visit 5, while a 1 L increment of FEV_1_ measure at Visit 5 was associated with −82.1 (95% CI: −104.0, −60.2) cm/s lower cfPWV at Visit 5. Similar patterns were seen when comparing prospective and cross-sectional associations of FVC with cfPWV. In further adjustment for covariates (Model 2), associations between lung function at Visits 1 and 5 with cfPWV at Visit 5 remained similar, although attenuated, and the association of FVC at Visit 1 with cfPWV was no longer statistically significant (Table 2). When minimally adjusted, a 1 L decrease in lung function over 20 years was associated with higher cfPWV for both FEV_1_ (β: 54.0; 95% CI: −2.9, 111.0) and FVC (β: 22.0; 95% CI: −28.9, 72.8), although results were not statistically significant at α=.05. Further adjustment for covariates attenuated associations of the 20-year decrease in lung function with cfPWV at Visit 5. Overall, the prospective, cross-sectional, and change in lung function associations were stronger between FEV_1_ and cfPWV at Visit 5 compared with the associations between FVC and cfPWV at Visit 5 (Table 2).

**Table 2.** The Minimally and Fully Adjusted, Predicted Difference in Carotid-Femoral Pulse Wave Velocity (cm/s) Prospectively Associated with a 1 L Greater Lung Function at Visit 1 and Cross-Sectionally at Visit 5, and the Minimally and Fully Adjusted, Predicted Difference in Pulse Wave Velocity (cm/s) Associated With a 1 L Decline in Lung Function Over 20 years, Using Multiple Imputation by Chained Equations to Impute Missing Exposure and Outcome in Participants That Were Alive During and Attended Atherosclerosis Risk in Communities Visit 5, (n = 5720).

	Model 1		Model 2	
	β estimate	95% CI	β estimate	95% CI
Lung function at Visit 1				
FEV_1_	−87.7	−105.0, −64.3	−26.3	−48.0, −4.7
FVC	−60.4	−77.1, −43.7	−13.6	−31.8, 4.6
Lung function at Visit 5				
FEV_1_	−82.1	−104.0, −60.2	−22.0	−45.8, 1.9
FVC	−66.3	−83.0, −49.7	−17.9	−37.5, 1.7
Decrease in lung function^a^				
FEV_1_	54.0	−2.9, 111.0	10.8	−46.2, 67.7
FVC	22.0	−28.9, 72.8	−4.4	−51.6, 42.8

Abbreviations: FEV_1_, forced expiratory volume in 1 second in liters; FVC, forced vital capacity in liters; cfPWV, carotid-femoral pulse wave velocity in cm/s; 95% CI: 95% confidence interval; β: the adjusted, predicted difference in pulse wave velocity (cm/s) associated with a 1 L difference in the lung function parameter.

Associations of lung function at Visit 1 and Visit 5 with continuous cfPWV assessed using multivariable linear regression. Associations of predicted 20-year decrease in lung function from Visit 1 to Visit 5 with continuous cfPWV assessed using linear mixed effects regression and linear regression models. Model 1 adjusted for height of the participant. Model 2 adjusted for race-center, sex, smoking status, age, time between measurements, height, BMI, high fasting plasma glucose, and relevant interaction terms.

^a^Mean centered decrease in lung function per 20 years.

### Associations of Lung Function and its Change with High Carotid-Femoral Pulse Wave Velocity

When assessing the association of lung function with dichotomous “high cfPWV,” patterns emerged similar to those described above. Prospective and cross-sectional minimally adjusted associations of lung function were not substantially different: a 1 L increment of FEV_1_ measure at Visit 1 was associated with lower odds of high cfPWV (odds ratio [OR]: .60; 95% CI: .52, .69), while a 1 L increment of FEV_1_ measure at Visit 5 was also associated with lower odds of high cfPWV (OR: .63; 95% CI: .54, .72). Similar patterns were observed when comparing the prospective and cross-sectional associations of FVC with cfPWV. When adjusted further for covariates (Model 2), prospective and cross-sectional associations of lung function with high cfPWV were attenuated and were no longer statistically significant at α = .05. In minimally adjusted models, a greater 20-year decrease in lung function appeared to be slightly associated with increased odds of high cfPWV, although this was not statistically significant and was brought essentially to the null when further adjusted for additional covariates. Greater FEV_1_ showed a slightly stronger association with lower odds of high cfPWV than FEV, although associations between FEV_1_ and FVC with cfPWV were more similar when adjusted for additional covariates (Table 3).

**Table 3.** The Adjusted, Predicted Odds of “High Carotid-Femoral Pulse Wave Velocity (cfPWV)” (Quartile 4 of cfPWV vs Quartiles 1-3) Prospectively Associated With a 1 L Greater Lung Function at Visit 1 and Cross-Sectionally at Visit 5, and the Adjusted, Predicted Odds of “High cfPWV” Associated With a 1 L Decline in Lung Function Over 20 years, Using Multiple Imputation by Chained Equations to Impute Missing Exposure and Outcome in Participants That Were Alive During and Attended Atherosclerosis Risk in Communities Visit 5, (n = 5720).

	Model 1		Model 2	
	OR	95% CI	OR	95% CI
Lung function at Visit 1				
FEV_1_	.60	.52, .69	.86	.73, 1.02
FVC	.68	.61, .76	.90	.79, 1.03
Lung function at Visit 5				
FEV_1_	.63	.54, .72	.89	.75, 1.06
FVC	.67	.60, .75	.89	.77, 1.04
Decrease in lung function^a^				
FEV_1_	1.39	.93, 2.08	1.04	.66, 1.63
FVC	1.18	.83, 1.68	.98	.68, 1.43

Abbreviations: FEV_1_, forced expiratory volume in 1 second in liters; FVC, forced vital capacity in liters; cfPWV, carotid-femoral pulse wave velocity in cm/s; OR, odds ratio; 95% CI, 95% confidence interval.

Referent group: quartiles 1-3 of cfPWV. “High cfPWV” defined as cfPWV = 1324-2855 cm/s. Associations of lung function at Visit 1 and Visit 5 with “high cfPWV” assessed using multivariable logistic regression. Associations of predicted decrease in lung function from Visit 1 to Visit 5 with “high cfPWV” assessed using linear mixed effects regression and logistic regression models. Model 1 adjusted for height of the participant. Model 2 adjusted for race-center, sex, smoking status, age, time between measurements, height, BMI, high fasting plasma glucose, and relevant interaction terms.

^a^Mean centered decrease in lung function per 20 years.

### Sensitivity Analyses

Inclusion of 3563 participants by MICE who met inclusion criteria, were alive at the time of ARIC Visit 5, but who did not attend the visit, did not alter inferences made from the main analyses. We did not find any differences in the associations by hypertension, smoking status, or high fasting plasma glucose.

## Discussion

Expiratory function (FEV_1_) and lung capacity (FVC) were prospectively and cross-sectionally inversely associated with central arterial stiffness and reduced odds of having high cfPWV later in life. In contrast, the 20-year rate of decline in lung function was not observed to be an important factor of this relationship. Although a higher rate of decline in lung function over 20 years was associated with having high cfPWV in later life when minimally adjusted for height, this association had wide confidence intervals, and the association was largely attenuated after further adjustment for CV risk factors. At both mid- and later-life, lower latter-life central arterial stiffness was more strongly associated with higher FEV_1_ than with higher FVC. These findings suggest that a relationship does exist between lung function and central artery stiffness across the adult life course and that mid-life lung function is predictive of later-life central artery stiffness.

Our finding that greater lung function was cross-sectionally associated with less stiff arteries largely supports previously published research.^17,19-22,24,25^ Bolton et al^19^ compared lung function in later-life with lung function 20 years earlier at mid-life in terms of associations with central arterial stiffness and concluded that lung function at mid-life was a better predictor of later-life arterial stiffness than measures of lung function later in life.^19^ However, our findings suggest that lung function at mid-life and later-life were both associated with later-life arterial stiffness. These conclusions should be interpreted with consideration of how differential attrition and missing data were dealt with, as the Bolton et al analysis did not account for loss to follow-up or missing data (∼25% of their baseline cohort), while our analysis used MICE to impute values for missing data among alive participants. Similar to our results, an investigation of expiratory function 5 years prior and at the same time as central artery stiffness measurement reported both prospective and cross-sectional associations.^17^ Okamoto et al examined decline in lung function over a 5-year period, reporting a larger decline to be associated with higher measures of arterial stiffness,^17^ whereas our study found largely no association between predicted 20-year decline in lung function and central arterial stiffness. It is conceivable that shorter-term changes in lung function are associated with arterial stiffness, but not long-term changes, since decline in lung function over the life course is a widely shared characteristic.^33^ Also to be considered, our statistical models were adjusted for smoking status, a large determinant of both lung function and arterial stiffness, whereas the analyses by Okamoto et al. were not.^17^

Although some previously mentioned studies have investigated pathophysiological processes that may link lung function and arterial stiffness, no causal mechanisms have been persuasively documented at this time. One of the mechanisms proposed to link lung function and central arterial stiffness involves systemic inflammation, which has been reproducibly related to chronic lung disease and impaired lung function, and is modestly associated with aortic stiffening.^17^ Whether systemic inflammation is antecedent to impaired lung function and central arterial remodeling, or rather a systemic response to inflammatory processes in these organs, remains unclear.^3^ Elevated blood pressure, along with cardiac structure and function, may also link lung function and central artery stiffening. Animal studies suggest that arterial stiffening precedes an increase in blood pressure, although the association may be bi-directional, while epidemiologic findings show that reduced lung function is predictive of increased systolic blood pressure later in life.^7,13^ Type 2 diabetes also has been considered a mediator of the association of poor pulmonary function and arterial stiffness. It has been proposed that reduced lung function is a risk factor for type 2 diabetes, whereas others have reported diabetes to be predictive of impaired lung function.^8^ Several studies on the other hand report a cross-sectional association between arterial stiffening and type 2 diabetes and suggest that central arterial stiffening may be a sequela of type 2 diabetes.^14^ A mediating role of diabetes has been posited through chronic low-grade systemic inflammation or autonomic neuropathy, both linked with diabetes, reduced lung function, and arterial stiffening.^14^ Alternatively, lung function decline and artery wall remodeling may merely co-occur with increasing age or share an unknown common cause.^33^

### Strengths and Limitations

One prior observational study investigated the association of age-related decline in lung function with later central arterial stiffness. Our study spans ∼20 years of change in lung function, rather than 5 years in the previously mentioned study, giving a broader picture of how lung function changes from mid-life into older age and its relationship with arterial stiffness measured at later life. Another strength of this study was the use of cfPWV, the gold standard measure of arterial stiffness, unlike several previously conducted cross-sectional studies.

One limitation of this study is the ∼20 year difference in lung function assessment time points without any intervening measurements. Further, changes in spirometry instrumentation required that the SensorMedics model 1022 dry-rolling seal volume spirometer be used at Visit 5, whereas the Collins Survey II water-seal spirometer was used at Visits 1 and 2. To our knowledge, the comparability of measurements from these two devices has not been documented, although studies comparing spirometry instruments indicate the main sources of variability to be the individual participants and technicians, with variability due to different instruments generally fixed and of small magnitude.^34^ A meta-analysis of spirometry measures from eight different cohort studies classified 88.4%, 91.2%, and 89.5% of ARIC spirometry measures as valid measurements (reproducibility of the two largest lung volumes within 150 mL) from visits 1, 2, and 5, respectively.^35^

As a further limitation, we were unable to assess changes in arterial stiffness concurrent with changes in lung function, as measures of arterial stiffness were not collected before Visit 5. Without knowing how arterial stiffness changed over 20 years in relation to changes in lung function, we are not able to assess whether these two factors concurrently degrade, or whether there is a causal relationship. Although we used MICE to reduce selection bias from differential attrition and missing data, a strength compared with studies that did not address these potential biases, selection bias may still remain. Attrition over the course of follow-up can be seen as related to somewhat poorer health at Visit 1 (Supplementary Table 1). Therefore, we anticipate that the estimates reported here likely underestimate the true association between lung function and central arterial stiffness, as those with the least favorable values of lung function were more likely to not attend Visit 5, or be deceased by that time.

## Conclusions

In this population-based cohort under extended follow-up, greater expiratory function and lung capacity measured both at mid-life and later-life were associated with lower degrees of central arterial stiffness in later life. A greater 20-year decline in lung function, however, was not associated with later-life arterial stiffness. These temporal relationships between lung function and central arterial stiffness contribute new information linking lung function and age-related central artery stiffening.

## Supplemental Material

Supplemental Material - Decline in Lung Function From Mid-to Late-Life With Central Arterial Stiffness: The Atherosclerosis Risk in Communities Study Click here for additional data file. Supplementary Material for Decline in Lung Function From Mid-to Late-Life With Central Arterial Stiffness: The Atherosclerosis Risk in Communities Study by Kennedy M. Peter, James R. Pike, John S. Preisser, Anna M. Kucharska-Newton, Michelle L. Meyer, Maria C. Mirabelli, Priya Palta, Timothy Hughes, Kunihiro Matsushita, Yifei Lu, and Gerardo Heiss in Angiology.
