# Supplementary material for: Decline in Lung Function From Mid-to Late-Life With Central Arterial Stiffness: The Atherosclerosis Risk in Communities Study
Source: Angiology. 2022 May 27;73(10):967–75. doi: 10.1177/00033197221105747 (PMC9490435; doi:10.1177/00033197221105747)
Supplement: Supplemental Material - Decline in Lung Function From Mid-to Late-Life With Central Arterial Stiffness: The Atherosclerosis Risk in Communities Study [file sj-pdf-1-ang-10.1177_00033197221105747.pdf]

Supplemental Figure 1. Flowchart of participant inclusion criteria.

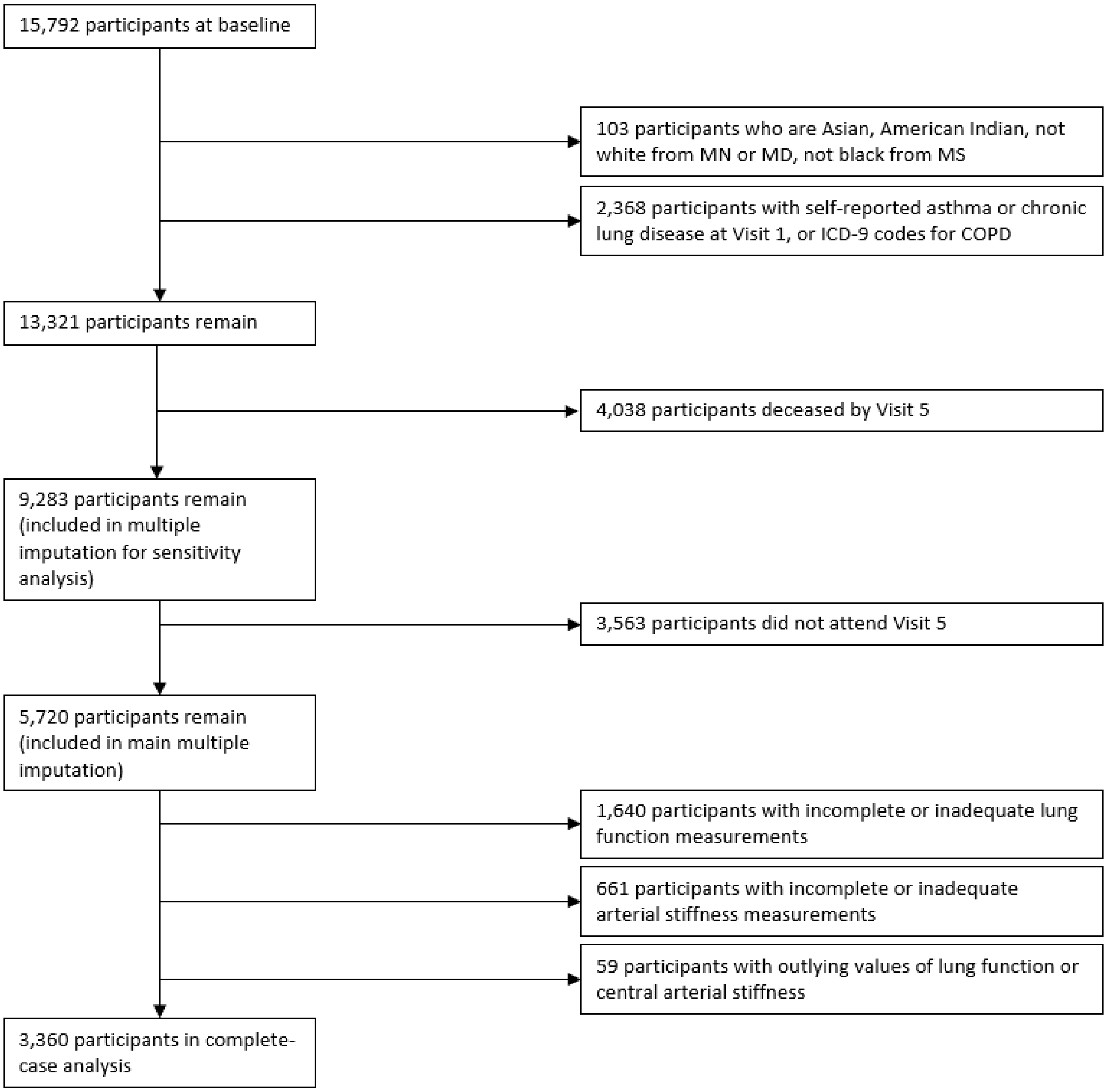

Supplemental Table 1. Demographic and health-related characteristics of the analytic sample at baseline, by vital status at ARIC Visit 5.

	Met inclusion criteria, no missing data	Missing/inadequate LF or cfPWV measurements	Alive, but did not attend Visit 5
	n (%)	n (%)	n (%)
Total analytic sample (n=9,283)	3,360 (36.2)	2,360 (25.4)	3,563 (38.4)
Study Center			
Forsyth County, NC	649 (19.3)	603 (25.5)	1,144 (32.1)
Jackson, MS	709 (21.1)	553 (23.4)	760 (21.3)
Suburbs of Minneapolis, MN	1,054 (31.4)	651 (27.6)	892 (25.0)
Washington County, MD	948 (28.2)	553 (23.4)	767 (21.5)
Female sex	2,007 (59.7)	1,355 (57.4)	2,173 (61.0)
Race			
Black	749 (22.3)	606 (25.7)	926 (26.0)
White	2,611 (77.7)	1,754 (74.3)	2,637 (74.0)
Age at Visit 1, mean (SD)	51.4 (4.9)	53.0 (5.4)	54.1 (5.7)
Body Mass Index at Visit 1, mean (SD)	26.5 (4.1)	28.2 (5.6)	27.8 (5.3)
High Fasting Plasma Glucose at Visit 1	130 (3.9)	195 (8.3)	314 (8.9)
Missing	21	19	33
Cigarette Smoking Status at Visit 1			
Current	498 (14.9)	412 (17.5)	795 (22.3)
Former	1,115 (33.2)	791 (33.6)	1,131 (31.8)
Never	1,744 (52.0)	1,155 (49.0)	1,634 (45.9)
Missing	3	2	3
Adjusted[1] FEV_1 at Visit 1, mean (SD)	3.1 (0.6)	2.9 (0.6)	2.8 (0.6)
Missing	0	321	510
Adjusted[1] FVC at Visit 1, mean (SD)	4.0 (0.8)	3.8 (0.8)	3.8 (0.8)
Missing	0	321	510
cfPWV at Visit 5, mean (SD)	1,161.2 (302.9)	1,230.8 (538.8)	---
Missing	0	1,053	3,563

FEV_1: forced expiratory volume in 1 second in liters; FVC: forced vital capacity in liters; SD: standard deviation; cfPWV: carotid-femoral pulse wave velocity in cm/s

[1]Excluding participants with inadequate spirometry measurements defined as meeting at least one of these conditions: data recording error (computer started after the start of expiration), breath-hold leak >5%, submaximal participant effort, spirogram not calibrated correctly, spirogram not reproducible; adjusted for age, height, sex, and race at Visit 1

Supplemental table 2. Complete case analysis of the minimally and fully adjusted, predicted difference in pulse wave velocity (cm/s) prospectively associated with a 1 L greater lung function at Visit 1 and cross-sectionally at Visit 5, and the minimally and fully adjusted, predicted difference in pulse wave velocity (cm/s) associated with a 1 L decline in lung function over 20 years, (N=3,360)

	Model 1		Model 2	
	β estimate	95% CI	β estimate	95% CI
Lung function at Visit 1				
FEV_1	-92.6	-113.6, -71.5	-39.6	-64.4, -14.8
FVC	-69.5	-86.2, -52.8	-28.6	-49.2, -8.0
Lung function at Visit 5				
FEV_1	-81.9	-103.6, -60.2	-28.3	-52.7, -3.9
FVC	-63.1	-80.2, -46.0	-22.0	-42.5, -1.4
Decrease in lung function*				
FEV_1	-55.3	-107.2, -3.5	-37.8	-88.9, 13.2
FVC	-29.8	-71.7, 12.1	-19.8	-60.6, 20.9

FEV_1: forced expiratory volume in 1 second in liters; FVC: forced vital capacity in liters; cfPWV: carotid-femoral pulse wave velocity in cm/s; 95% CI: 95% confidence interval; β: the adjusted, predicted difference in pulse wave velocity (cm/s) associated with a 1 L difference in the lung function parameter

*Mean centered decrease in lung function per 20 years

Associations of lung function at Visit 1 and Visit 5 with continuous cfPWV assessed using multivariable linear regression.

Associations of predicted 20-year decrease in lung function from Visit 1 to Visit 5 with continuous cfPWV assessed using linear mixed effects regression and linear regression models.

Model 1 adjusted for height of the participant.

Model 2 adjusted for race-center, sex, smoking status, age, time between measurements, height, BMI, high fasting plasma glucose, and relevant interaction terms.

Supplemental table 3. Complete case analysis of the adjusted, predicted odds of “high cfPWV” (quartile 4 of cfPWV vs. quartiles 1-3) prospectively associated with a 1 L greater lung function at Visit 1 and cross-sectionally at Visit 5, and the adjusted, predicted odds of “high cfPWV” associated with a 1 L decline in lung function over 20 years, (N=3,360)

	Model 1		Model 2	
	OR	95% CI	OR	95% CI
Lung function at Visit 1				
FEV_1	0.52	0.44, 0.62	0.79	0.64, 0.97
FVC	0.60	0.52, 0.68	0.81	0.68, 0.97
Lung function at Visit 5				
FEV_1	0.59	0.50, 0.70	0.87	0.71, 1.08
FVC	0.65	0.57, 0.74	0.88	0.74, 1.05
Decrease in lung function*				
FEV_1	0.65	0.43, 0.96	0.77	0.50, 1.19
FVC	0.77	0.56, 1.06	0.86	0.61, 1.21

Referent group: quartiles 1-3 of cfPWV

FEV_1: forced expiratory volume in 1 second in liters; FVC: forced vital capacity in liters; cfPWV: carotid-femoral pulse wave velocity in cm/s; OR: odds ratio; 95% CI: 95% confidence interval

“High cfPWV” defined as cfPWV = 1324-2855 cm/s

*Mean centered decrease in lung function per 20 years

Associations of lung function at Visit 1 and Visit 5 with “high cfPWV” assessed using multivariable logistic regression.

Associations of predicted decrease in lung function from Visit 1 to Visit 5 with “high cfPWV” assessed using linear mixed effects regression and logistic regression models.

Model 1 adjusted for height of the participant.

Model 2 adjusted for race-center, sex, smoking status, age, time between measurements, height, BMI, high fasting plasma glucose, and relevant interaction terms.
